# Neurophysiological Changes Induced by Chronic *Toxoplasma gondii* Infection

**DOI:** 10.3390/pathogens6020019

**Published:** 2017-05-17

**Authors:** Ellen Tedford, Glenn McConkey

**Affiliations:** Heredity, Disease & Development, Faculty of Biological Sciences, University of Leeds, Leeds LS2 9JT, UK; bs10et@leeds.ac.uk

**Keywords:** *Toxoplasma gondii*, neurophysiology, host-parasite interaction, neuroimmune, testosterone, dopamine, catecholamine, glutamatergic

## Abstract

Although the parasite *Toxoplasma gondii* is one of the most pervasive neurotropic pathogens in the world, the host-parasite interactions during CNS infection and the consequences of neurological infection are just beginning to be unraveled. The chronic stages of infection have been considered dormant, although several studies have found correlations of infection with an array of host behavioral changes. These may facilitate parasite transmission and impact neurological diseases. During infection, in addition to the presence of the parasites within neurons, host-mediated neuroimmune and hormonal responses to infection are also present. *T. gondii* induces numerous changes to host neurons during infection and globally alters host neurological signaling pathways, as discussed in this review. Understanding the neurophysiological changes in the host brain is imperative to understanding the parasitic mechanisms and to delineate the effects of this single-celled parasite on health and its contribution to neurological disease.

## 1. Introduction

The concept of the ”extended phenotype” describes how the expression of an organism’s genes affect not only that organism, but may have a wide reaching impact [1]. In the case of *Toxoplasma gondii*, mechanisms induced by parasitic genes can lead to neurophysiological changes that alter host behavioral changes and can facilitate the life cycle of the parasite.

The loss of fear phenotype is well characterised: chronically infected rodents no longer respond to cat odour with fear and indeed the physical response is reversed to attraction [2]. It is surmised that in the wild this results in increased rodent predation and facilitate the spread of the parasite to *Felidae*, the definitive host of *T. gondii* wherein all sexual lifecycle stages take place [3]. However, the molecular mechanisms underlying the loss of fear phenotype are poorly delineated. Humans are considered “accidental hosts” as they do not perpetuate the parasite life cycle. However, there is no evidence that *T. gondii* differentiates between intermediate hosts and it is likely to exert similar neurophysiological effects on all intermediate hosts, including humans. Given the ubiquity of *T. gondii* in the human population, it is essential to understand these neurophysiological changes and the parasite induced cellular mechanisms that cause them as they have many potential far-reaching clinical consequences.

*T. gondii* is one of the world’s most pervasive parasites, infecting a wide variety of mammals that act as intermediate hosts. Infection can occur through congenital passage, consumption of oocysts that are present in the environment, or tissue cysts present in infected meat. Although precautions can be made to prevent infection (see the Centers for Disease Control www.cdc.gov/parasites/toxoplasmosis/), there is a risk of accidental infection, especially for children. Initial acute infection is transient, characterized by the presence of tachyzoites [4]. Toxoplasmosis, only observed in the immunocompromised or those infected while in utero, is caused by unchecked, rapidly dividing tachyzoites. Recently published data has demonstrated tachyzoites are able to cross the blood-brain barrier via infection and lysis of barrier endothelial cells [5]. After approximately three weeks, the host’s immune system controls tachyzoite growth, by which time some parasites have differentiated into bradyzoite developmental stages. The bradyzoites become encysted, forming the chronic stage of infection that can persist for the lifetime of the host. Historically considered a latent stage and only associated with pathogenesis in the immunocompromised host, recent data has demonstrated that bradyzoites continue to replicate [6,7]. However, as the host-parasite interactions are delineated, the chronic stages have become more clinically significant [8].

## 2. Host Behavioral Consequences

*T. gondii* infection is associated with altered neurological functioning. Indicators of motor function such as response times, memory, and co-ordination are reduced during chronic human and rodent infection [9,10,11]. Chronic *T. gondii* infection in the human population has been correlated with a diverse range of human diseases, including Alzheimer’s and Huntington’s [12,13]. The association between *T. gondii* infection and mental health disorders such as depression, psychosis, self-directed violence, and schizophrenia have been widely studied [14,15,16,17,18]. Schizophrenia is the most extensively investigated of these. Indeed, two meta-analyses of the association collectively spanning 70 studies over 55 years concluded that there is a positive correlation between *T. gondii* seropositivity and schizophrenia [19,20]. Despite this evidence, countries with a high incidence of *T. gondii* seroprevalence do not have corresponding increased schizophrenia incidence [21].

Given the diverse range of disorders associated with infection, it is clear that the neurophysiological changes induced are exceptionally complex. Unravelling the many mechanisms involved in the host-parasite interaction is essential to further understand mammalian neurobiology and why *T. gondii* is correlated with several human diseases.

## 3. Neuro-Immune Response

The immune response to this pathogen is an obvious potential contributor to neurological and behavioral changes. The brain is principally considered an immune-privileged tissue as it lacks a lymphatic system and tight capillary junctions within the blood-brain barrier prevent diffusion of large molecules, limiting lymphocyte access to the organ. This provides limited shelter from the host immune response for a pathogen, yet responses to infection are still measurable and necessary to suppress reactivation of chronic stages [22,23]. Chronic *T. gondii* infection is characterized by increased levels of host immune activity and neuroinflammation [24]. Evidence suggests that in the mouse model, T cell recruitment peaks at approximately 30 days post-infection and subsequently decreases [25].

Molecular initiators of the immune response to chronic infection include pathogen-associated molecular patterns (PAMPs) such as *T. gondii* profilin, which is able to act as a ligand for the toll-like receptor-11 (TLR11) [26]. This elicits a robust response from TLR11^+^ dendritic cells, inducing the MyD88 dependent activation of nuclear factor-κB (NF-κB), and up-regulating expression of pro-inflammatory cytokines such as tumor necrosis factor-α (TNF-α), interleukin-12 (IL-12), and interleukin-1β (IL-1β) [27]. *T. gondii* infected MyD88^−/−^ mice were found to have reduced IL-12 and interferon-γ (INF-γ) in the sera compared to wild type controls and high mortality. TLR11^−/−^ mouse models exhibited greater immunopathology and natural killer cell associated interferon-γ. They also had an increased tissue cyst burden, implying that this pathway plays an important role in regulating the chronic phase of infection [26,28].

IL-12 also plays an important role in the recruitment of natural killer (NK) cells and T cells [29]. Indeed, SCID mice combatted *T. gondii* infection with a NK cell mediated INF-γ response [30]. Microglia and to a lesser extent macrophage are important producers of INF-γ and crucial for cell mediated protection. Mice and humans with mutant alleles in genes of the IFN-γ signalling pathway are unable to suppress bradyzoite activation and are extremely susceptible to infection. Once released, IFN-γ can diffuse throughout the brain and bind to cell surface receptors, inducing the JAK/STAT signalling pathway, which results in phosphorylated STAT1 and increased transcription of immunity-related GTPases, the transcription factor IRF1, and major histocompatibility complex class II molecules [31].

INF-γ also stimulates the release of indoleamine 2,3-dioxygenase (IDO), catabolising tryptophan [32]. Indeed, *T. gondii* requires tryptophan and limiting tryptophan concentration is a mechanism by which parasite growth can be restricted [33]. Tryptophan is the precursor to serotonin and, hence, increased IDO concentration during infection may reduce synthesis of this neurotransmitter. Products of tryptophan catabolism kynurenic acid and quinolinic acid in the brain were found to increase oxidative stress, damaging cells, and eventually leading to apoptosis [34]. Kynurenic acid is an antagonist for glutamate ionic receptors and is able to inhibit α7 nicotinic acetylcholine receptors, modulating dopaminergic and glutamatergic neurotransmission [35]. Elevated kynurenic acid is also observed in the brains of schizophrenia and bipolar sufferers [36,37]. Chronic *T. gondii* infection induces early activation of tryptophan metabolism and kynurenic acid production; as observed in a Huntington’s disease mouse model [13]. A seven-fold increase in kynurenic acid was found in the brain of infected animals [38].

Cerebral immune responses are likely to be a compounding factor during infections, augmenting predisposition to mental health disorders such as depression [39]. This is likely to be particularly true for infections of the central nervous system. However, other neurotropic infections that induce a chronic immune response, such as chronic infections with cytomegalovirus and meningitis, are not associated with the specific behavioral changes observed with *T. gondii* infection. Based on these, it remains unclear how a chronic immune response could induce the specific behavioral and neurophysiological changes observed during *T. gondii* infection. Indeed, the specific “fatal feline” attraction and other behavioral changes are only observed following an established chronic *T. gondii* infection, typically with *T. gondii* infection 60 days post infection, at which point the host immune response is weakest [40]. The loss of fear phenotype was even observed in mice infected with an attenuated *T. gondii* strain after clearance of the parasite and when no immune response was detectable [41].

## 4. Hormonal Changes with Chronic Infection

Parasite induced changes to the host endocrine system provide another possible mechanism to alter host behavior and induce the “fatal feline” attraction phenotype. There have been observable sex differences regarding host changes in response to *T. gondii* infection. However, the majority of studies have been performed with male rodents, biasing much of our knowledge regarding the involvement of hormones. One example of sex specific effects on behavior is the observation that female rats exhibit the loss of fear of cat odor phenotype, except during estrus [42,43].

Induction of the host immune response could concertedly function with activating hormonal changes in the host. Host stress responses induced by a chronic immune response can activate the hypothalamic-pituitary-adrenal (HPA) axis, regulating homeostasis in the main body systems [44]. Activation of this pathway increases blood glucocorticoid concentration. Elevated glucocorticoid concentration is associated with neurodegeneration and synapse regression [45]. Furthermore, increased glucocorticoid concentration is correlated with many of the same disorders as *T. gondii* infection including schizophrenia and Alzheimer’s disease [46,47]. Activation of the HPA axis is a promising candidate to serve as a contributing factor to the changes with *T. gondii* infection. However, evidence linking this to host behavioral change is lacking. 

Altered testosterone levels have been observed with *T. gondii* infection, although the literature lacks consensus. Evidence suggests that testosterone activation may cause sexual arousal, directed towards feline odour in some rodents [48]. Interestingly, if male rats have been castrated prior to infection, they do not exhibit the loss of fear phenotype, suggesting that testosterone plays a direct role in this behavior [49]. Both testosterone and activation of the HPA axis are able to stimulate release of arginine vasopressin, a neurotransmitter associated with reproductive behavior. In chronically infected rats hypomethylation of the arginine vasopressin gene promoter region was observed in the amygdala [50]. Published data has reported both increased and decreased testosterone associated with *T. gondii* seropositivity in humans [51,52,53].

## 5. Neurophysiological Changes

Tropism for a particular brain region remains a straightforward explanation for host behavioral manipulation. However, the published data does not support this model and there is a lack of consensus in the literature (reviewed in [54]). In the largest study to date, in which 109 rats were infected by the natural route with oocysts, only mild tropism for the colliculus was found and there was a high degree of variability [55]. Furthermore, random distribution of tissue cysts was found within the forebrains of 45 chronically infected rats [56]. Given this, there is a paucity of evidence to correlate cyst location with host behavioral changes, with low cyst burden evident in many infected animals. This raises the important question, how can low numbers of cysts located disparately in brains of different hosts elicit the same host behavioral responses? Given current evidence, a plausible explanation is that *T. gondii* is able to induce host manipulation independent of tissue cyst location.

Although tissue localization is not supported, there is clear evidence of *T. gondii* tropism for neurons in which encysted parasites were exclusively found [57,58]. This is not due to selective invasion, as cultured primary explants find *T. gondii* infection in astrocytes, microglia, andneurons [59]. Hence, there may be properties of neurons that promote cyst development. This highlights the point that a considerable amount about neuron-parasite interactions is unknown. What is the mechanism by which the parasite is able to recognize neuronal cells or the property of neurons that promotes cyst development? Further, are specific neuronal sub-populations preferentially infected? Studies in neuronal cells and the recent development of stem cell cultures may shed light on these questions [60].

The morphology of neurons is altered by tissue cysts. 3D imaging analysis found cysts predominantly in neuronal processes such as dendritic spines rather than soma [61]. The formation of these, up to 70 μm in diameter and increasing in size as infection progresses, may reduce neuronal functionality [6]. Indeed, dendritic spine length and density of infected mice was significantly reduced compared to uninfected control animals [62,63]. Interestingly, dendritic spine loss and loss of dendritic function is also associated with schizophrenia and other mental health disorders [64]. It should be noted that initial infection, mediated by rapidly dividing tachyzoites, can cause extensive neuronal damage. However, behavioral changes have almost exclusively been associated with the chronic stage of infection. A narrative of purely physical local impairment randomly located is difficult to reconcile with the specific altered phenotypes associated with *T. gondii*.

## 6. Neurotransmitter Changes

The functional capacity of infected neurons is still largely unknown. There is growing evidence that rather than inhibiting the functionality of neurons, *T. gondii* is able to subvert their functions. In the 1970s, Stibbs et al. originally described changes in neurotransmitter concentration with infection, reporting a reduction in serotonin and norepinephrine, and an increase in total dopamine in the brains of chronically infected mice [65]. Since then, several publications have found altered neurotransmitter regulation with *T. gondii* infection. Recently published findings observed increases in dopamine turnover in chronically infected mice [66]. Ihara et al. found an increase in dopamine metabolites and a reduction in norepinephrine and serotonin [67]. Prandovszky et al. also observed elevated levels of total dopamine and metabolite content in infected catecholaminergic cells [68,69]. It has been observed in vitro that supplemental dopamine is able to increase parasite proliferation [70]. Treatment of rats and mice with dopamine receptor antagonists inhibit the establishment of behavioral changes with *T. gondii* infection [71,72]. Intracellular dopamine that is not properly packaged into vesicles can cause cell damage via free radical generation and this, or alternative mechanisms (e.g., neuroimmune responses, host gene regulation), may contribute to dendritic spine damage observed with *T. gondii* infection [73].

Current evidence suggests that observed changes in cerebral catecholamine concentration is due to altered synthesis rather than a relocation of surrounding dopamine. Infection did not alter levels of host tyrosine hydroxylase, DOPA decarboxylase (DDC), or the vesicular monoamine transporter (VMAT) in catecholaminergic cells, although DDC was observed in the parasitophorous vacuole in vitro and within tissue cysts in vivo [68]. Catecholamine dysregulation may also be affected by disruption of catabolism of dopamine (e.g., this would be coherent with the observed reduction in norepinephrine concentration with infection). A reduction in norepinephrine concentration is implicated in a variety of movement disorders in humans. This provides a possible explanation for the association between toxoplasmosis and movement disorders [74,75].

Although dopamine antagonist treatment blocked behavior changes, it has not yet been demonstrated that disrupting catecholamine synthesis can reverse observed behavioral phenotypes with infection. Gaskell et al. found that the *Toxoplasma* genome contains two copies of a gene encoding an enzyme with tyrosine hydroxylase activity, the rate limiting enzyme in the synthesis of l-3,4-dihydroxyphenylalanine (L-DOPA), precursor to dopamine [76]. The in vivo function of this enzyme in *T. gondii* is still under investigation, and a double knock-out of both genes has yet to be achieved. Combined with the observation of host DDC localising to bradyzoite cysts, this provides a possible explanation for observed elevated dopamine synthesis in dopaminergic cells in the absence of changes in host tyrosine hydroxylase [68]. Xiao et al. observed down-regulation of D1 type dopamine receptors (DRD1, 5) and dopamine metabolizing enzyme MaoA with infection [77]. An miRNA involved in regulating neuronal function, dopamine signalling and synaptic transmission, miRNA-132 expression was also down-regulated during chronic infection [78,79,80]. Hence, multiple factors may be altered that together result in elevated dopamine neurotransmission. This is coherent with observations of a blunted response to amphetamine treatment in locomotor tests in infected animals [81].

Glutamate signalling in the brain may also be “altered” with infection. Recent data has observed increased extracellular glutamate with chronic infection and a two-fold reduction in expression of the glutamate transporter (GLT-1) in glial cells [82]. The changes may be induced as a component of the neuroimmune responses to infection. Glutamate signalling is regulated by γ-aminobutyric acid (GABA), an inhibitory neurotransmitter that may also play an important role during infection. In infected animals, the GABAergic pathway remained intact, although global changes in localization of GAD67, the enzyme responsible for converting glutamate to GABA, were observed [83]. Interestingly, although only a small number of tissue cysts were found in infected mice, GAD67 location was disturbed throughout the brain. Increases in glutamate and/or disruption of GABAergic signaling could produce neurophysiological consequences such as seizures that are associated with *T. gondii* infection [84].

## 7. Parasite Secreted Effector Molecules

There is growing evidence that alterations to host cellular function may be induced by parasite-secreted factors. Host-parasite communication initiated by rhoptry (ROP) proteins injected during the tachyzoite infection continue after the formation of the parasitophorous vacuole. During infection, host-parasite communication is partly mediated by effector molecules secreted from parasite dense granules (GRA proteins) that are trafficked to the host cell cytosol and nucleus [85]. Recently, GRA24 was observed to subvert host cell MAP kinase pathways, altering host immune responses and allowing the parasite to survive undetected [86]. It remains possible that a secreted parasite derived protein such as a ROP or GRA protein may invoke host behavioral changes though altering host neurophysiology.

Groundbreaking work by Koshy et al. has demonstrated that *T. gondii* is able to inject rhoptry proteins into cells it does not infect [87]. Parasite derived proteins are able to regulate the host immune response (comprehensive review can be found at [88]). For example, *T. gondii* dampened INF-γ stimulated JAK/STAT1 responses and ROP16 induced STAT3/6 phosphorylation; blocking parasite induced inflammatory responses [89,90]. Furthermore, ROP16 altered IL-12 mediated responses of infected macrophage [91], whereas ROP18 phosphorylated p65, a subunit of NF-κB, targeted it for cellular degradation and suppressed the host immune response [92]. Given that tissue cysts are only found in a limited number of neurons, a parasite-secreted factor would provide a mechanism for exerting global effects. The injection of parasite proteins by circulating parasites could impose these global effects, although this phenomenon has yet to be observed with bradyzoites.

## 8. Epigenetic Changes

Epigenetic manipulation of the host genome is emerging as a new frontier of host-parasite interaction. Recent publications have observed infection induced chromatin changes in the host. Dass et al. found altered DNA methylation of the vasopressin receptor in the amygdala of chronically infected mice [50]. Altered methylation status of the promoters of spermatogenesis genes in infected testis was also observed [93]. This suggests that *T. gondii* is able to co-op host mechanisms of chromatin regulation. Advances in epigenetics have demonstrated that DNA methylation within cells is not as restricted to development as was once considered: DNA methylation is a dynamic process [94,95,96]. Epigenetic modifications are essential for neurological functions such as memory and hormonal and behavioral responses. Many neurological diseases correlated with *T. gondii* seroprevalence are the product of complex genetic and environmental factors. For example, schizophrenia, self-directed harm, and depression are associated with neuronal DNA methylation changes [97,98,99]. Understanding this complex interplay between genetic predisposition, *T. gondii* as an epigenator, and environmental factors is crucial in order to delineate the *T. gondii* extended phenotype.

Further research is important to discern epigenetic changes in the host in response to infection, as this may provide some explanation for the stability and consistency of behavioral changes between hosts. An epigenetic basis for behavioral alterations could explain the persistence of the loss of fear phenotype observed in mice that were infected with an attenuated *T. gondii* strain even after clearance of the parasite and when no immune response was detectable [41].

## 9. Future Directions

**Figure 1:**
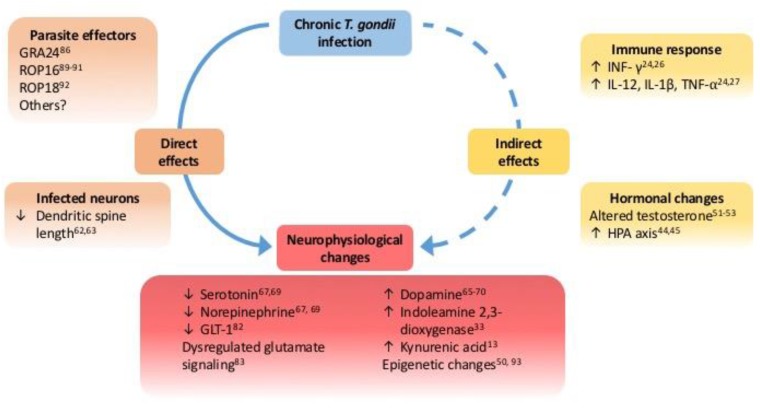
Directly and indirectly mediated effects of chronic *T. gondii* infection on host neurophysiology. Model of mechanisms involved with host responses to infection (i.e., neuroimmune and hormonal changes) indirect and more likely confounding factors, augmenting neurophysiological changes rather than inducing them. Indeed, the specificity of behavioral changes associated with infection suggest that direct mechanisms of the parasite-host interaction play a significant role in the neurophysiological changes associated with chronic *T. gondii* infection.

Although evidence to correlate *T. gondii* infection to a variety of human diseases is growing, there remains a paucity of understanding about the molecular mechanisms involved and the alterations to neurophysiology. Changes in host cells, both to those infected and exposed to infection, require full characterization particularly during the cyst stages of infection within neurons. We do not yet understand the full extent of neurophysiological changes induced by *T. gondii* infection and how these alter host behavior. As molecular and genomic tools advance, mapping changes in the host induced by *T. gondii* will be essential to understanding how an individual may be predisposed to a range of disorders. There are also possible GxE interactions between specific susceptible genotypes (e.g., genes linked to neurological disease such as Disc1) and infection as an environmental factor that need further exploration. A recent study found that mice with a mutation in Nurr1, which regulates mesoaccumbens and mesocortical dopamine levels, had higher open field activity when infected with *T. gondii* [100]. Advances in the area of stem cell cultures and ex vivo tissue imaging systems (i.e., CLARITY) will allow visualisation of tissue cysts within transparent neuronal tissue [101]. Understanding the neuron-parasite interaction is essential to understand the mechanisms used by this unicellular parasite to induce the complex extended phenotype observed. A summary of the possible direct and indirect mechanisms and neurophysiological changes induced by chronic *T. gondii* infection is shown (Figure 1).

## 10. Conclusions

In light of major programs to understand functionality of the brain in “healthy” and sick individuals, elucidating the neurophysiological consequences of this ubiquitous parasite are crucial. Indirect effects of infection such as the host mediated immune and hormonal response are likely to be contributing factors to host behavioral change. However, they are unconvincing as the primary effectors of change. Chronic neuro-immune and HPA axis activation may further induce analogous effects. However, it is unclear how they mediate the specific behavioral phenotypes associated with infection [102,103]. Parasite-mediated changes such as the epigenetic changes observed can induce specific changes in host cells. Very specific subtle behavioral changes can be associated with global modifications in neurotransmission. For example, common schizophrenia medications inhibit dopamine D2 receptors. These act globally, affecting catecholamine signaling on neurons expressing the receptors, yet these are beneficial, specifically effecting the positive symptoms of schizophrenia, although some side-effects are observed [104]. This suggests that the relationship between neurophysiological changes and behavior is not linear.

Current evidence suggests that host neuronal activity is altered or subverted by chronic *T. gondii* infection, leading to an extended phenotype wherein host behavior is permanently changed. *T. gondii* infection presents a unique model by which we can understand the complexity of human neurophysiology. By discovering the complex mechanisms exploited by *T. gondii*, we will gain insight into not only the host-parasite interaction but mammalian neurophysiology.
